# Apoptosis-based therapy to treat pulmonary arterial hypertension

**Published:** 2016

**Authors:** Yuichiro J. Suzuki, Yasmine F. Ibrahim, Nataliia V. Shults

**Affiliations:** 1 Department of Pharmacology and Physiology, Georgetown University Medical Center, Washington DC 20007 USA; 2 Department of Pharmacology, Minia University School of Medicine, Minia, Egypt

**Keywords:** Anti-tumor drugs, Apoptosis, Cancer chemotherapeutic agents, Pulmonary arterial hypertension, Pulmonary hypertension, Right heart failure, Right ventricle, Vascular remodeling

## Abstract

Pulmonary arterial hypertension (PAH) is rare, but patients who are diagnosed with this disease still suffer from a lack of satisfactory treatment strategies to prolong survival. While currently approved drugs for PAH have some benefits, these vasodilators only have limited efficacy for eliminating pulmonary vascular remodeling and reducing mortality. Thus, our laboratory has been exploring the use of aggressive drugs, which are capable of causing apoptotic cell death, to treat PAH. We have so far found that three classes of anti-tumor agents, including anthracyclines, taxanes, and proteasome inhibitors, are capable of reducing pulmonary vascular thickness in rats with PAH. These drugs kill cells in remodeled pulmonary vessels without affecting the normal, healthy pulmonary vasculature, revealing that proliferating vascular cells in PAH patients are more sensitive to drug-induced apoptosis compared to the differentiated phenotype that is physiologically important for smooth muscle contraction. Since many apoptosis-inducing drugs cause cardiotoxicity in cancer patients, and because PAH patients already have a weakened heart, we focus on finding biological mechanisms that may reverse pulmonary vascular remodeling without promoting cardiotoxicity. We found two agents, dexrazoxane and pifithrin-α, that selectively inhibit cardiac muscle apoptosis without affecting the drug-induced apoptosis of the proliferating pulmonary vascular cells. Thus, we propose that the addition of apoptosis-inducing drugs and cardioprotectants to PAH therapies may be effective in treating patients and preventing right heart failure.

## Introduction

Pulmonary arterial hypertension (PAH) is a rare disease with prevalence estimated between 15 and 50 cases per million people^1^. PAH prevalence is two-to-four times higher in women than in men^2,3^, and the disease can affect people of any age, even children. The early symptoms of PAH include mild dyspnea, fatigue, and dizziness, which can also be associated with various other diseases. Further, accurate diagnosis of PAH requires an invasive right-heart catheterization^2^. As a result, PAH diagnosis is often delayed and, by the time patients are diagnosed with PAH, the disease is progressed to the occurrence of pulmonary vascular remodeling and right ventricle (RV) remodeling^3^.

In PAH, elevated pulmonary vascular resistance causes stress to the RV, and RV failure is the major cause of death in patients with PAH^4^. The narrowing and occlusion of pulmonary vessels, as well as the elevation of pulmonary vascular resistance are due to multiple factors, including vasoconstriction and the thickening of pulmonary vascular walls. Currently approved drugs for PAH treatment primarily cause vasodilation, but these have limited influence on the structural changes in the vessel walls.

**Figure 1:**
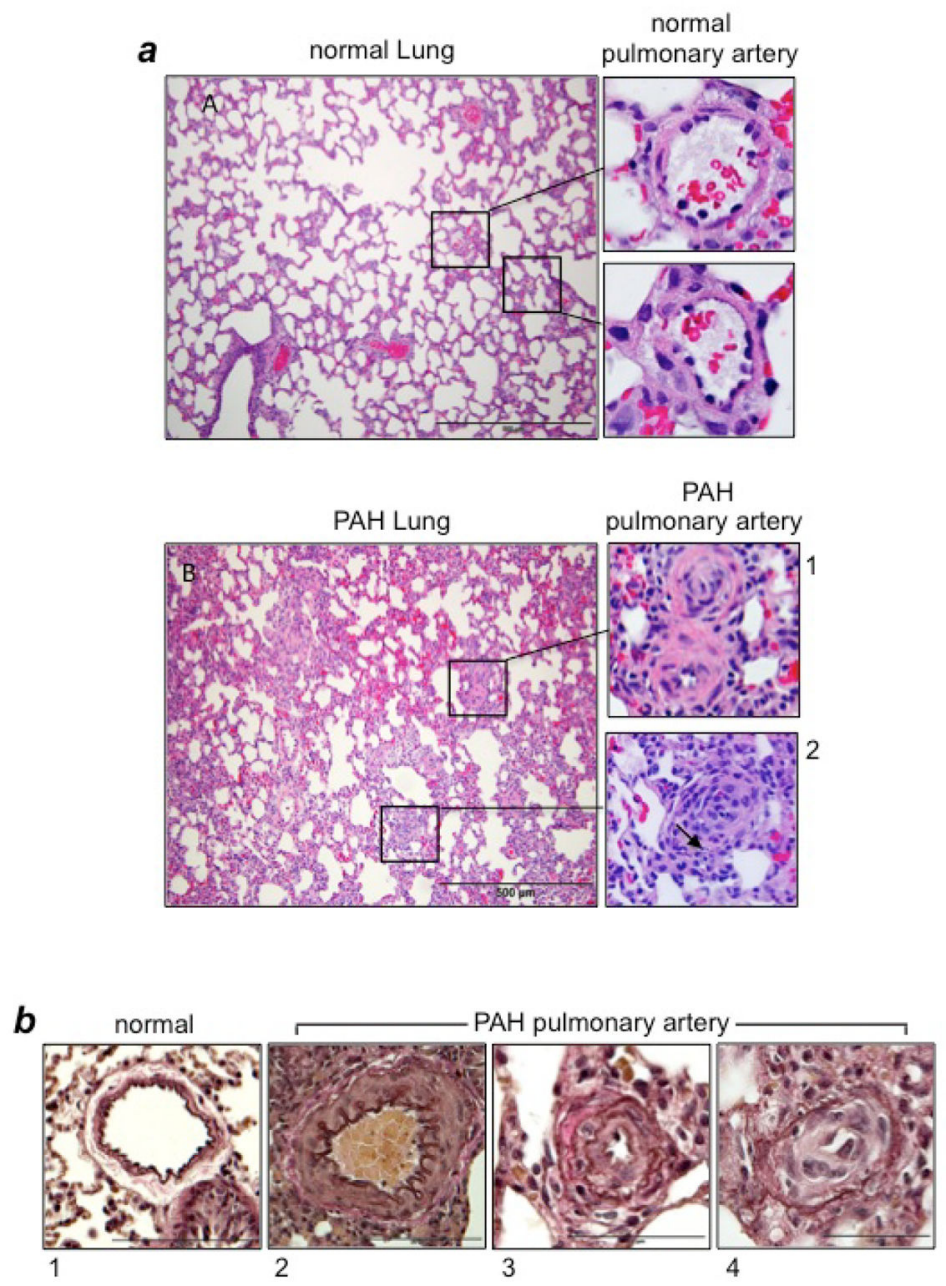
Vascular lesions in PAH (a) Hematoxylin and eosin (H&E) stain of the lung and pulmonary artery (PA) from normal control rats and rats with PAH induced by SU5416/hypoxia^12,20^. In PAH lungs, atelectasis and emphysematous expansion of alveoli with thickened alveolar septa are seen. Around the vessels, perivascular inflammatory infiltrates are present. Panel 1 shows occluded small PAs in rats with PAH. Panel 2 shows the occluded central artery and a rounded collection of endothelial cells forming small channels (arrow). (b) Verhoeff-Van Gieson stains of PAs. Panel 1 shows thin intima and muscular media in normal control rats. By contrast, PAs of rats with PAH induced by SU5416/hypoxia^12,20^ exhibit hypertrophy and hyperplasia of medial smooth muscle cells and an increased amount of collagen fibers (Panel 2); the intimal thickening due to endothelial cell proliferation and the destruction of the inner elastic lamina (Panel 3), and intimal proliferation characterized by onion-skin-like layers with narrow lumens and no shirring of elastic membranes (Panel 4).

Increased pulmonary vascular wall thickness and the narrowing and, in some cases occlusion, of the pulmonary arterial lumens are due to the growth of pulmonary arterial cells such as smooth muscle cells, endothelial cells, and fibroblasts, which form various lesions including medial hypertrophy; eccentric intimal proliferation; concentric, non-laminar intimal thickening; concentric, laminar intimal thickening; fibrinoid necrosis; occlusive intimal proliferation; and plexiform legions. Figure 1 exhibits some pulmonary vascular lesions in PAH with narrow or occluded blood vessel lumens that would significantly limit blood flow. Thus, therapeutic strategies to inhibit cell-growth signaling should prevent the formation and delay the progression of pulmonary vascular remodeling.

However, as noted above, PAH patients are often diagnosed after developing severe changes in the pulmonary vasculature, suggesting that inhibiting cell growth may have only limited effects in terms of reducing vascular resistance. Thus, our laboratory is exploring the effects of therapeutic agents that are capable of causing the death of pulmonary vascular cells in order to eliminate lesions that contribute to the narrowing and occlusion of the pulmonary vessels (Figure 1). Since it is preferable to kill unwanted cells in a controlled fashion, inducing programmed cell death, such as apoptosis, may be a fruitful avenue. Though, we refer to this approach as “apoptosis-based therapy,” the mechanisms of programmed cell death could include those other than apoptosis, as discussed below.

Research in cancer therapy, which also frequently utilizes apoptosis-based approach, has demonstrated that one major problem with using cell death-causing agents is that they can also cause the deaths of cells which we do not want to kill. Notably, many anti-tumor drugs cause the death of cardiac myocytes, exerting cardiotoxicity^5^. Since PAH patients already suffer from RV dysfunctions, the use of such drugs, which could potentially worsen the cardiac problems in PAH patients, poses challenges. In this regard, over the last decade, our laboratory has been investigating: (i) whether pulmonary vascular cells are susceptible to apoptosis; (ii) whether the administration of apoptosis-inducing, anti-tumor drugs reverses pulmonary vascular remodeling; and (iii) what the effects of these drugs are on RVs influenced by PAH.

## Effects of Anti-Tumor Drugs on Pulmonary Vascular Remodeling

**Figure 2:**
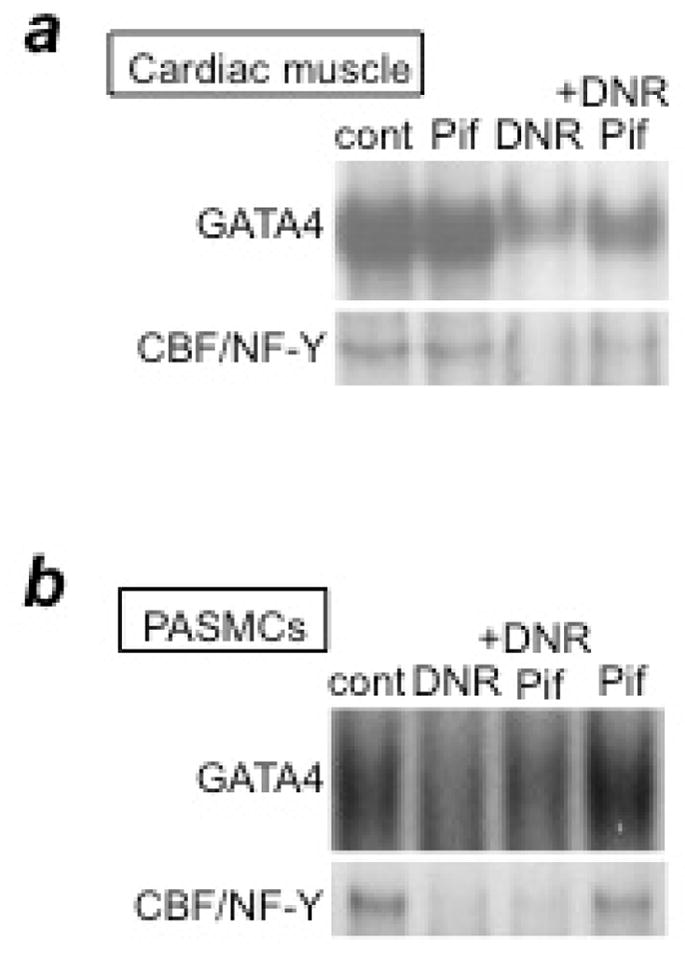
p53 inhibition by pifithrin-α blocks DNR-induced downregulation of GATA4 and CBF/NF-Y in cardiac muscle cells, but not in PASMCs (a) Cardiac muscle cells or (b) PASMCs were pre-treated with pifithrin-α (Pif) and then treated with DNR. Nuclear extracts were prepared and the DNA-binding activities of GATA4 and CBF/NF-Y were monitored by electrophoretic mobility shift assays.

In considering which anti-tumor agents should be first studied in order to develop apoptosis-based therapy for PAH, we decided to use daunorubicin (DNR). DNR is the first member of the anthracycline class of anti-tumor agents and has been used clinically for 50 years. One reason we investigated this drug is that DNR was found to be an effective inducer of apoptosis in cultured human pulmonary artery smooth muscle cells (PASMCs)^6^. Another important reason was that since anthracyclines have cardiotoxic properties, particularly left ventricle (LV) toxicity, in cancer patients^5^, such drugs may serve as an excellent model for understanding how normal RVs and RVs affected by PAH may be influenced by anti-tumor drugs. While the RV and LV are generally thought to have identical molecular characteristics, our studies have revealed differential molecular-signaling mechanisms in the two ventricles^7,8^. Thus, we thought we might find that the RV is not as susceptible as the LV to the toxic effects of anthracycline administration. Further, our studies using cultured cells revealed that the mechanisms of DNR-induced cell death processes differ between PASMCs and cardiac muscle cells^9^. We reported that DNR kills cardiac muscle cells via p53-dependent inhibition of the DNA-binding activity of the CBF/NF-Y transcription factor toward the CCAAT box within the promoter region of *Gata4* that regulates the gene transcription of anti-apoptotic proteins^10^. In cardiac muscle cells, a p53 inhibitor, pifithrin-α, suppresses DNR-induced CBF/NF-Y activity and therefore GATA4 downregulation and cell death (Figure 2a). By contrast, in PASMCs, while DNR did inhibit GATA4 and CBF/NF-Y DNA-binding activities, the inhibition of p53 by pifithrin-α had no effects (Figure 2b). These results suggest that p53 inhibitors protect the heart without influencing the ability of DNR to kill PASMCs.

To determine the effect of DNR in intact animals, Sprague-Dawley rats were exposed to chronic hypoxia for 2 weeks to develop pulmonary hypertension and pulmonary vascular remodeling, characterized by thickening of the smooth muscle mass. After pulmonary hypertension and vascular thickening were developed, DNR was administered. We found that, within 3 days, DNR at a concentration as low as 5-mg/kg body weight significantly reduced pulmonary arterial wall thickness, a reduction associated with increased apoptosis^11,12^. Importantly, neither the reduction of vascular wall thickness nor the induction of apoptosis occurred in the pulmonary vessels of normal control rats, suggesting that remodeled pulmonary vasculature is sensitive to DNR-induced cell killing.

We found that proteasome inhibitors -- bortezomib and MG-132 -- also reversed pulmonary vascular remodeling in chronic hypoxia-induced pulmonary hypertensive rats^12,13^. Bortezomib has been used to treat multiple myeloma and relapsed mantle cell lymphoma.^14^ To produce the more severe pulmonary vascular remodeling, resembling the human disease, rats were injected with SU5416 (inhibitor of the vascular endothelial growth factor receptor tyrosine kinase) and then subjected to chronic hypoxia for 3 weeks^12,15,16^. Subsequently, maintaining these rats in normoxia for 2 weeks developed medial as well as intimal thickening in the pulmonary vasculature, and proteasome inhibition effectively reduced vascular wall thickness in this model^12^.

## Possible Mechanism for the Increased Susceptibility of Remodeled Pulmonary Vasculature to Cell Killing

As described above, DNR reduced the thickness of remodeled pulmonary arterial walls in rats exposed to chronic hypoxia without affecting the pulmonary vessels of normoxic control rats. Understanding this remarkable mechanism should help in developing improved therapeutic agents for PAH.

**Figure 3:**
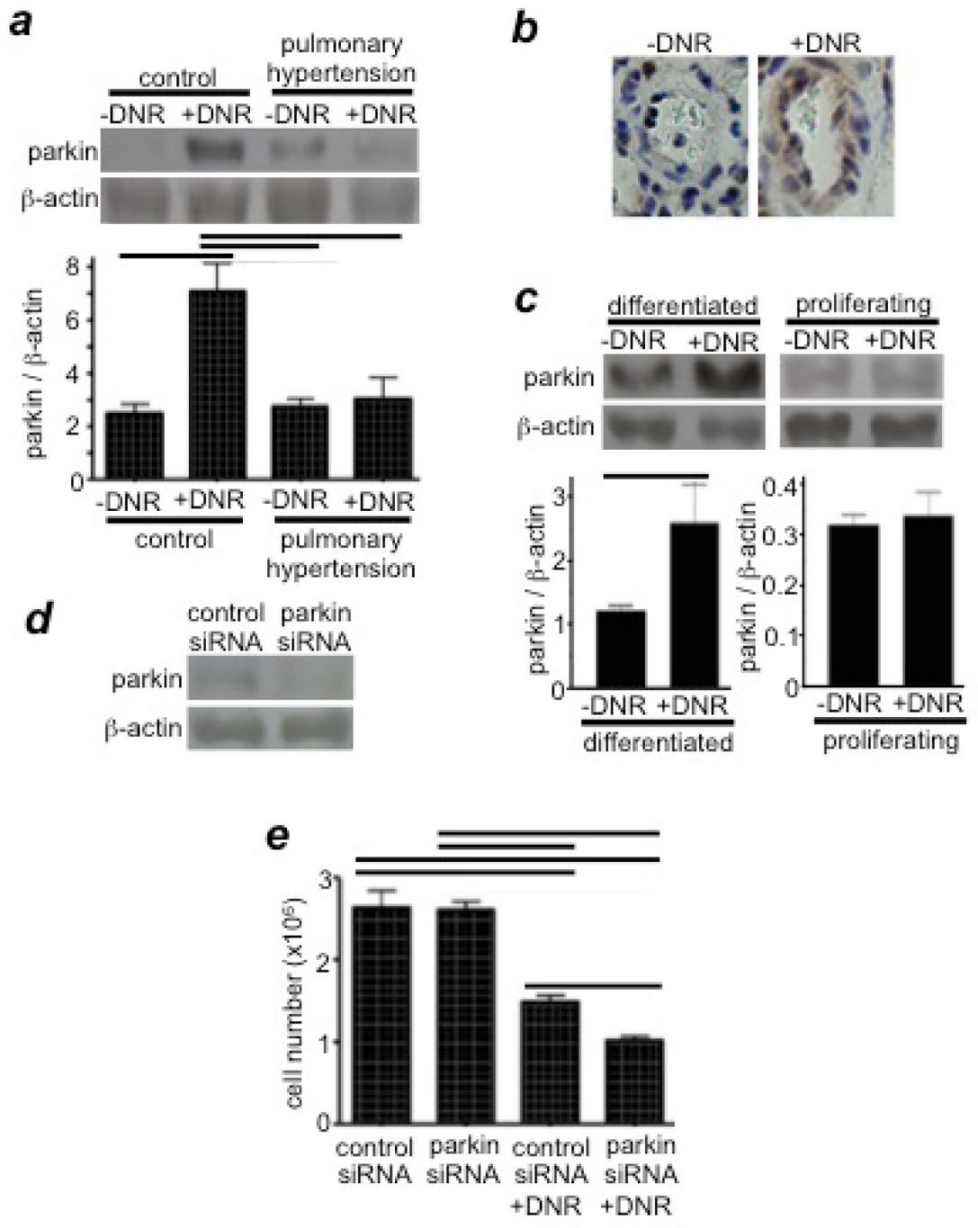
Parkin regulates drug-induced cell death (a) Rats were exposed to hypoxia for two weeks to develop pulmonary hypertension, and were then injected with DNR (5 mg/kg body weight). Rats were then placed back in hypoxia. One day after the injection, isolated pulmonary arteries were homogenized, and parkin levels were monitored by Western blotting. Reproduced from Ibrahim et al.^12^ (b) Parkin expression levels were monitored by immunohistochemistry using the parkin antibody. (c) Differentiated and proliferating cultured human PASMCs were treated with DNR for 5 h. Cell lysates were prepared, and parkin levels were monitored by Western blotting. Reproduced from Ibrahim et al.^12^ (d) The extent of parkin knockdown by transfecting human PASMCs with control or parkin siRNA. (e) Human PASMCs transfected with control or parkin siRNA were treated with DNR, and the cell number was determined by counting on a hemocytometer. Reproduced from Ibrahim et al.^12^ Bar graphs represent means ± SEM. Bars denote the values significantly different from each other at P<0.05.

Our experiments suggested that parkin, an E3 ubiquitin ligase that plays an important role in Parkinson’s disease^17^, is also expressed in PASMCs and may define the mechanism for the increased susceptibility of remodeled pulmonary vascular walls to DNR-induced cell death. We found that DNR increases the expression of parkin in the pulmonary arteries of normal rats, but not in rats with PAH (Figures 3a & 3b)^12^. Similarly, DNR only increased parkin expression in cultured human PASMCs that had been differentiated to have the contractile phenotype, but not in proliferating cells (Figure 3c)^12^. Thus, in normal contractile PASMCs, parkin may suppress DNR-induced cell killing. Indeed, parkin seems to function as a cell survival factor in PASMCs, as siRNA knockdown of parkin exaggerated DNR-induced cell killing (Figures 3d & 3e)^12^. Future investigations along this line should shed light on an approach to most effectively target unwanted remodeled pulmonary vascular cells.

## Anti-Tumor Drugs and Cardiotoxicity in PAH

**Figure 4:**
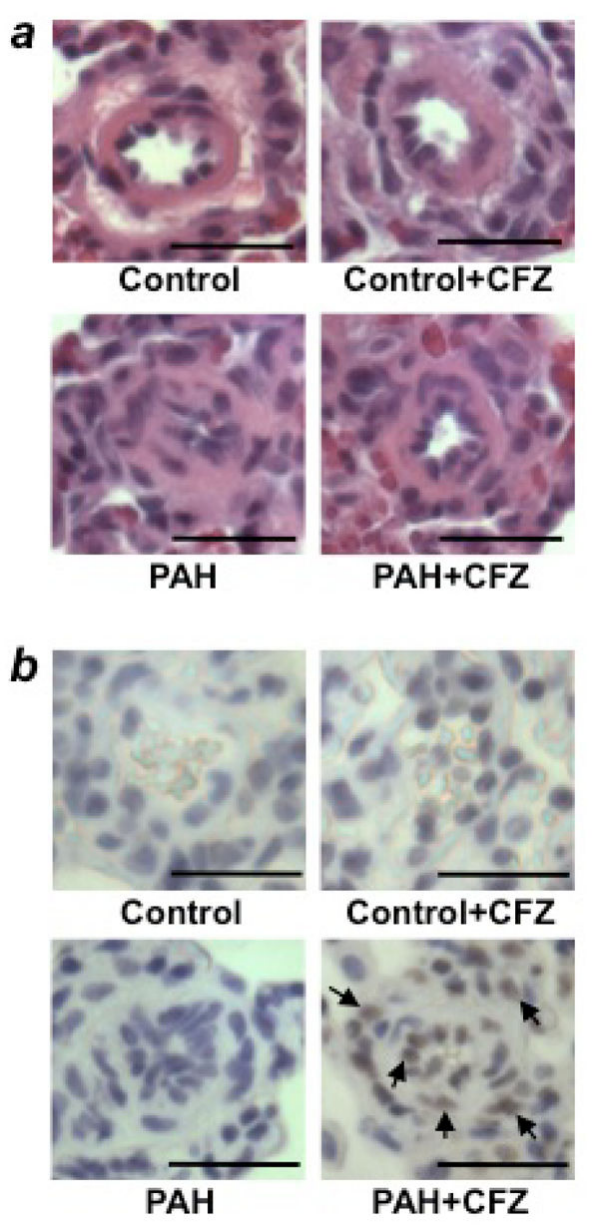
CFZ reduces the wall thickness of remodeled pulmonary arteries and promotes apoptosis without affecting normal pulmonary arteries in SU5416/hypoxia model of PAH Rats were injected with SU5416 and exposed to hypoxia for 3 weeks, and then placed in normoxia for 5 weeks. Rats were then injected with 6 mg/kg body weight CFZ, twice per week for 2 weeks. The rats were sacrificed after 3 days of the last injection. (a) Representative H&E staining of small pulmonary arteries (diameters ranging from 50μm to 100μm). Scale bars, 50 μm. (b) Representative TUNEL assay results. Scale bars, 50 μm. Reproduced from Wang et al.^20^

Our study noted no apparent cardiotoxicity, such as the induction of apoptosis by DNR (5 mg/kg body weight), in the hypertrophied RV^12^. However, since bortezomib was reported to produce cardiotoxicity in animals with pulmonary hypertension^18^, we explored the effects of carfilzomib (CFZ), considered to be a safe and effective alternative to bortezomib in the treatment of multiple myeloma^19^. In two rat models of PAH (SU5416/hypoxia and SU5416/ovalbumin), CFZ reduced pulmonary vascular wall thickness and induced programmed cell death (Figure 4)^20^. Like DNR, the effects of CFZ were only observed in the pulmonary vasculatures of rats with PAH, but not in normal control rats. While the remodeled RVs were not susceptible to CFZ-induced apoptosis, significant apoptosis was observed in the LVs of animals with PAH. To find possible cardioprotective agents, in accordance with the cell-culture experiments described above, pifithrin-α (a p53 inhibitor) was administered along with CFZ in rats with PAH. Remarkably, pifithrin-α completely protected the LV against CFZ-induced apoptosis. Moreover, pifithrin-α did not affect the action of CFZ to induce the apoptosis of pulmonary vascular cells or to reduce vascular wall thickness in rats with PAH^20^. Thus, p53 inhibitors protect the heart against drug-induced toxicity without altering the efficacy of antitumor agents to reverse pulmonary vascular remodeling. While we do not yet understand the mechanism, a clinically used, FDA-approved cardioprotectant against anti-tumor agents, dexrazoxane, also exhibited protective effects against CFZ-induced cardiotoxicity in PAH rats without affecting the efficacy of CFZ to reverse pulmonary vascular remodeling^20^. Thus, including dexrazoxane or pifithrin-α should be considered during apoptosis-based therapy to treat PAH using anti-tumor drugs.

## Targeting the RV Damage Promoted by PAH in the Treatment Strategy

**Figure 5:**
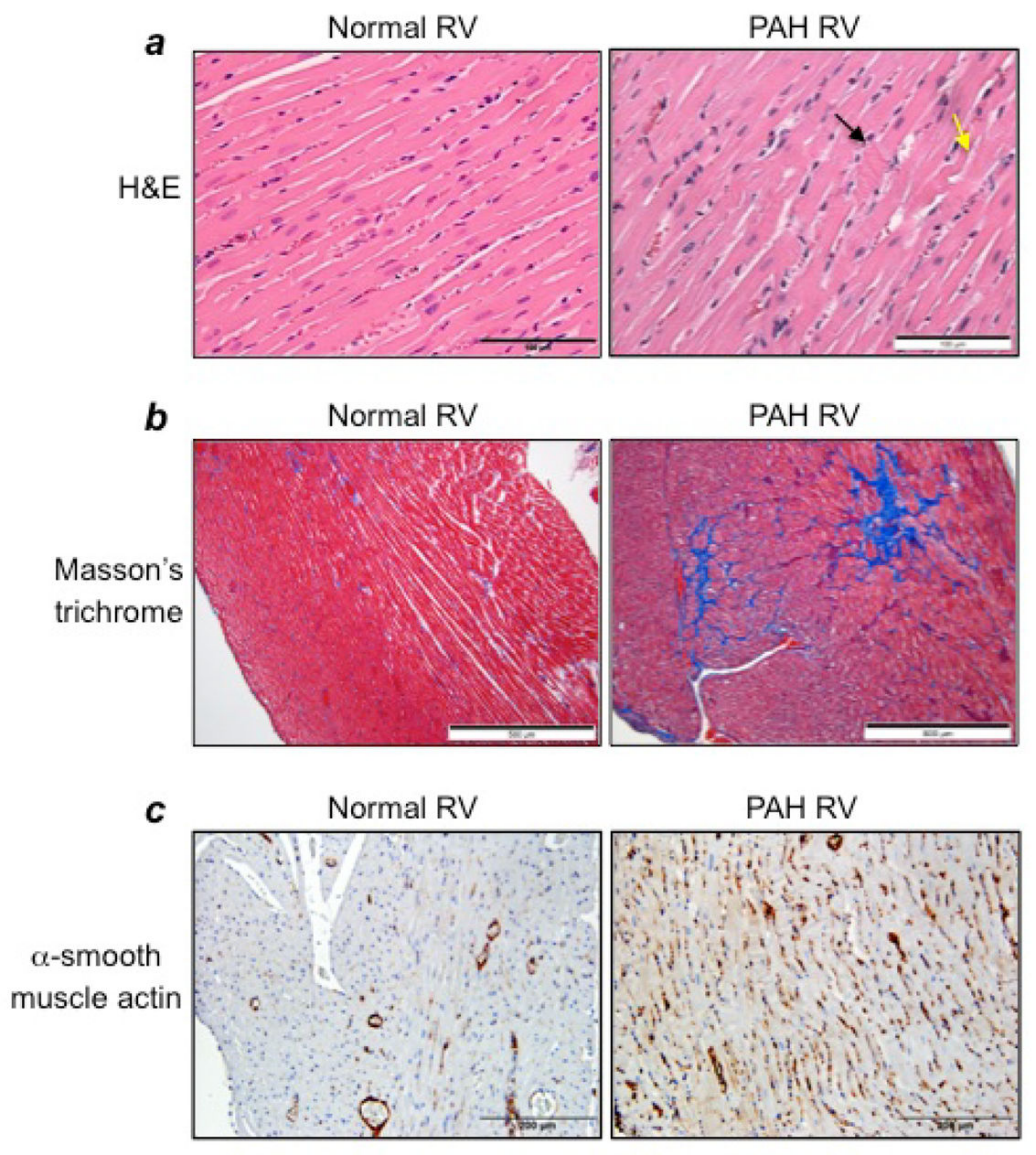
Characteristics of the RV in PAH Rats were subjected to SU5416/hypoxia treatment, and then hearts were fixed in 10% formalin and sectioned for histology analyses. (a) H&E stain (Magnification ×400), (b) Masson’s trichrome staining (Magnification ×100), (c) Immunohistochemistry with α–smooth muscle actin antibody (Magnification ×200).

Pulmonary hypertension per se does not kill patients; rather, right heart failure is the major cause of death among PAH patients. In addition to pulmonary vascular remodeling and severe narrowing and occlusion of the pulmonary vascular lumen, by the time patients are diagnosed with PAH, severe RV modifications have already occurred, including the death of RV myocytes as well as the induction of RV fibrosis. Therefore, a treatment that can preferentially kill remodeled pulmonary vascular cells without affecting normal cells and also reduce mechanisms that contribute to these RV dysfunctions would be ideal. As shown in Figure 5a, in response to PAH, the RV suffers from major damage to the myocardium. H&E stain shows the cardiomyocytes degeneration; polymorphic and hypertrophied cardiomyocytes; myofiber disarray; hypereosinophilia and hyperbasophilia of cardiomyocytes; contractures of cardiomyocytes (black arrow); myocytolysis (yellow arrow); a loss of myofiber striation; wavy arrangement of myofiber; edema of the cytoplasm; and indistinct nuclear borders within cardiomyocytes. A dramatic increase in the levels of collagen was also demonstrated in the RVs of PAH animals, as shown by the blue stain in Masson’s trichrome-stained RV sections (Figure 5b). These changes in the RVs of PAH animals are also associated with the increased expression of α-smooth muscle actin (Figure 5c), indicating the formation of myofibroblasts in the RVs of rats with PAH. Thus, the treatment strategy that can kill cardiac fibroblasts and/or myofibroblasts without affecting cardiomyocytes and activate collagen-degrading systems such as matrix metalloproteinases would be desirable. Subsequently, eliminated fibrotic areas need to be replaced by functional myocardium, perhaps through the formation of cardiomyocytes from resident cardiac progenitor cells. For example, isl1-positive cells that serve as progenitors of the secondary heart field from which the RV is derived during development^21^ may contribute to the restoration of RV myocytes. The inclusion of agents that can activate these mechanisms in the treatment strategies would help repairing the damaged RV in PAH patients.

## Summary and Future Perspectives

Currently, no therapeutic agents are capable of satisfactorily prolonging the lives of patients who are diagnosed with PAH, perhaps because currently available therapies have only limited effects on the pulmonary vascular remodeling that is an important aspect of this disease. Histologic observations in humans and experimental animals show dramatic narrowing and occlusion of the pulmonary vascular lumens, clearly indicating limited blood flow. Thus therapeutic strategies to reverse the narrowing and occlusion of the pulmonary vascular lumen should be necessary aspects of the treatment of PAH. In rat models of PAH, our laboratory observed that some cell-death-inducing anti-tumor drugs reverse pulmonary vascular remodeling without influencing the normal pulmonary vasculature. Further, the perception that giving these drugs to PAH patients is contraindicated appears to be incorrect, as remodeled RVs were found to be more resistant to damage by these drugs, and we discovered agents that are capable of protecting the heart without reducing the anti-tumor drug’s efficacy in the pulmonary vasculature. Collectively, these data generated using animal models of PAH suggest that combining cell-killing drugs and cardioprotectants with the currently available vasodilator therapies may present optimal therapeutic strategies to treat this lethal disease. Clinical studies to test this concept are warranted. Successfully curing PAH, however, depends on future studies that use experimental models to define exactly which anti-tumor agents (either alone or in combination) should be given via which route of administration, which doses, and under which timing or duration of treatment. Further, possible unwanted effects of such treatment regimes, such as drug resistance, should also be investigated.
